# Association Studies with Imputed Variants Using Expectation-Maximization Likelihood-Ratio Tests

**DOI:** 10.1371/journal.pone.0110679

**Published:** 2014-11-10

**Authors:** Kuan-Chieh Huang, Wei Sun, Ying Wu, Mengjie Chen, Karen L. Mohlke, Leslie A. Lange, Yun Li

**Affiliations:** 1 Department of Biostatistics, University of North Carolina, Chapel Hill, North Carolina, United States of America; 2 Department of Genetics, University of North Carolina, Chapel Hill, North Carolina, United States of America; 3 Department of Computer Science, University of North Carolina, Chapel Hill, North Carolina, United States of America; National Taiwan University, Taiwan

## Abstract

Genotype imputation has become standard practice in modern genetic studies. As sequencing-based reference panels continue to grow, increasingly more markers are being well or better imputed but at the same time, even more markers with relatively low minor allele frequency are being imputed with low imputation quality. Here, we propose new methods that incorporate imputation uncertainty for downstream association analysis, with improved power and/or computational efficiency. We consider two scenarios: I) when posterior probabilities of all potential genotypes are estimated; and II) when only the one-dimensional summary statistic, imputed dosage, is available. For scenario I, we have developed an expectation-maximization likelihood-ratio test for association based on posterior probabilities. When only imputed dosages are available (scenario II), we first sample the genotype probabilities from its posterior distribution given the dosages, and then apply the EM-LRT on the sampled probabilities. Our simulations show that type I error of the proposed EM-LRT methods under both scenarios are protected. Compared with existing methods, EM-LRT-Prob (for scenario I) offers optimal statistical power across a wide spectrum of MAF and imputation quality. EM-LRT-Dose (for scenario II) achieves a similar level of statistical power as EM-LRT-Prob and, outperforms the standard Dosage method, especially for markers with relatively low MAF or imputation quality. Applications to two real data sets, the Cebu Longitudinal Health and Nutrition Survey study and the Women’s Health Initiative Study, provide further support to the validity and efficiency of our proposed methods.

## Introduction

Genotype imputation has become standard practice in modern genetic studies [1] [2] [3] [4]. For each untyped variant imputed, standard imputation methods estimate posterior probabilities of all possible genotypes. For example, when the untyped variant is bi-allelic with alleles A and B, we obtain posterior probabilities for A/A, A/B, and B/B with the constraint of summation being one. Such probability information can be further summarized into degenerate one-dimensional summary statistics including the mode (the best guess genotype, or the genotype with the highest posterior probability), or the mean (the imputed dosage).

Since association analysis with phenotypes of interest rather than genotype imputation per se is usually of the ultimate interest, development and evaluation of post-imputation association strategies have therefore attracted considerable attention from the research community [5] [6] [7] [8] [9] [10] [11] [12] [13]. Among them, imputation dosage based methods provide an attractive compromise between modeling complexity, computational efficiency and statistical power, have been shown analytically to be optimal among methods based on one-dimensional summary statistics [11], and thus have been most commonly adopted in recent imputation-aided genome-wide association studies (GWAS) and meta-analyses [14] [15] [16] [17]. On the other hand, explicitly modeling the probabilities of all possible genotypes using the mixture of regression models (abbreviated Mixture hereafter and detailed below) has the best performance in terms of statistical efficiency, particularly with low imputation quality, but at the cost of increased computational complexity [13].

Limited evaluations of existing methods (including methods that explicitly model posterior probabilities) on variants with low imputation quality suggest much reduced power compared with accurately imputed variants, for instance, as demonstrated in Figure 2 and 3 [13] and Figure 2 [11]. Analysis of variants with low imputation quality is not surprisingly a challenging problem due to the low correlation between imputed and true genotypes. It is nevertheless an increasingly important problem because as sequencing-based reference panels continue to grow [18] [19], we have increasingly more well imputed markers but also even more markers with relatively low imputation quality, particularly at markers with lower allele frequencies [20] [21] [22] [23] [24]. It is thus highly warranted to seek alternative and potentially more efficient methods to model imputation uncertainty for these markers. In this work, we develop expectation-maximization likelihood-ratio tests (EM-LRT) that can accommodate either posterior genotype probabilities, when available (EM-LRT-Prob), or imputed dosages (EM-LRT-Dose). Simulations and real data application demonstrate the validity of the proposed methods and suggest them as a computationally more efficient alternative to the best existing method (Mixture) for association analysis of variants with low MAF or imputation quality.

**Figure 1:**
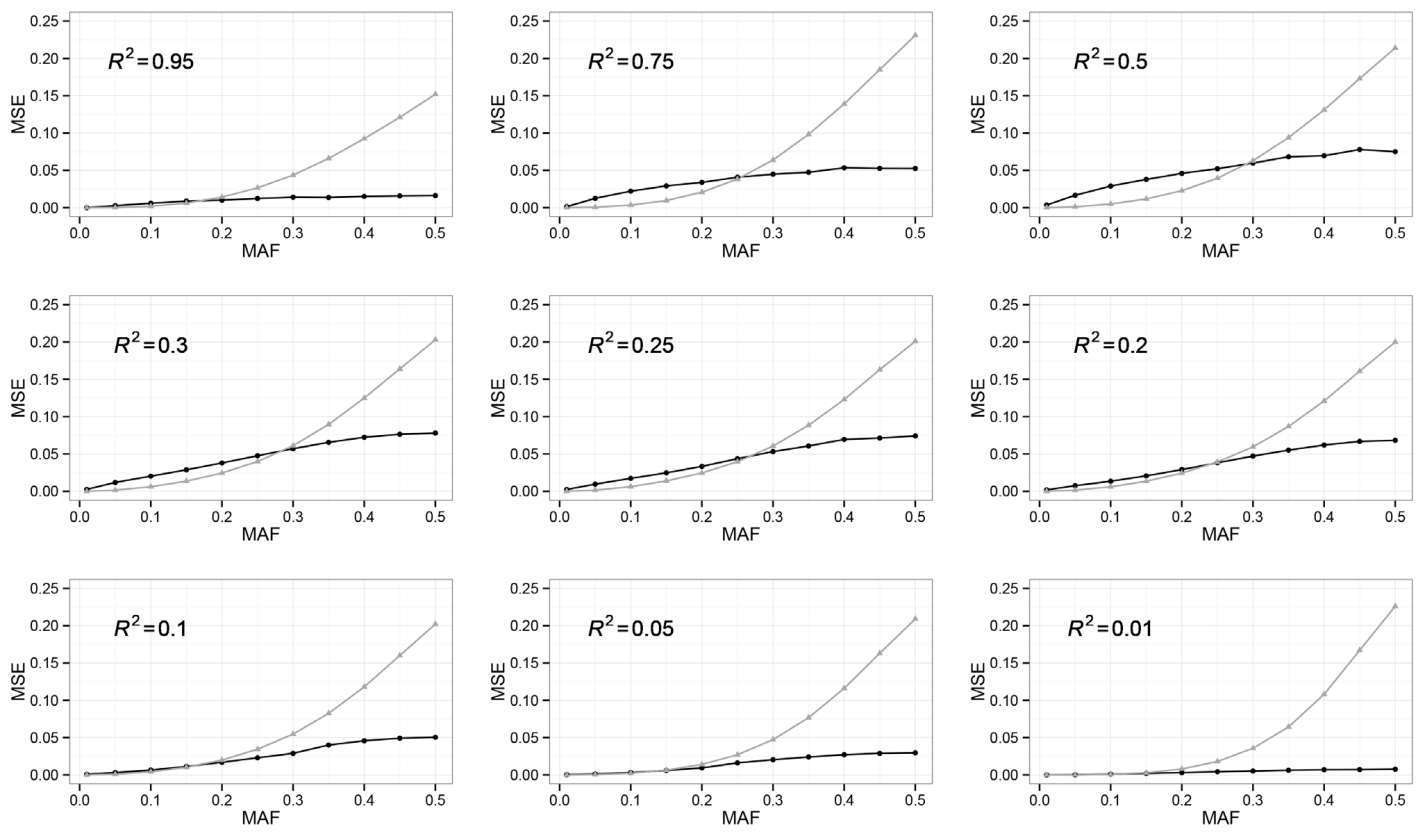
MAF Threshold: Rejection Sampling (Black) vs. Dosage Approximation (Grey). The MSE (Y-axis) between sampled genotype probability 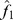 and true 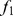 using rejection sampling (black) and dosage approximation (grey) is compared across a spectrum of *R^2^*.

**Figure 2:**
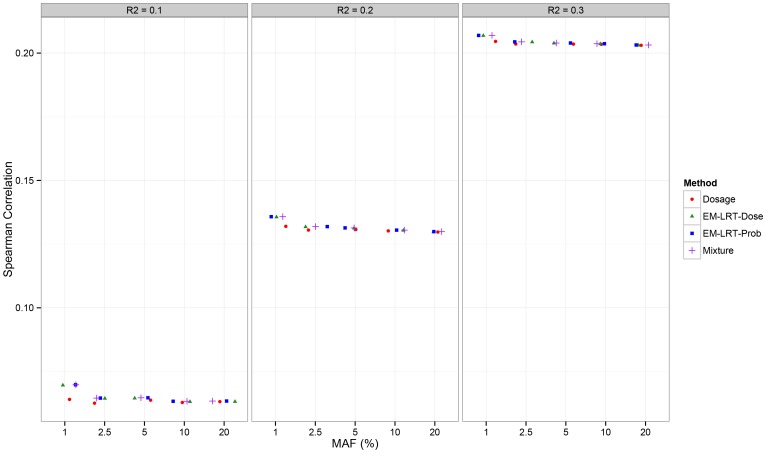
Spearman Correlation with Gold Standard *P*-values. The Spearman correlation (Y-axis) between gold standard *p*-values and *p*-values from different methods is displayed across a spectrum of MAF and *R^2^*.

**Figure 3:**
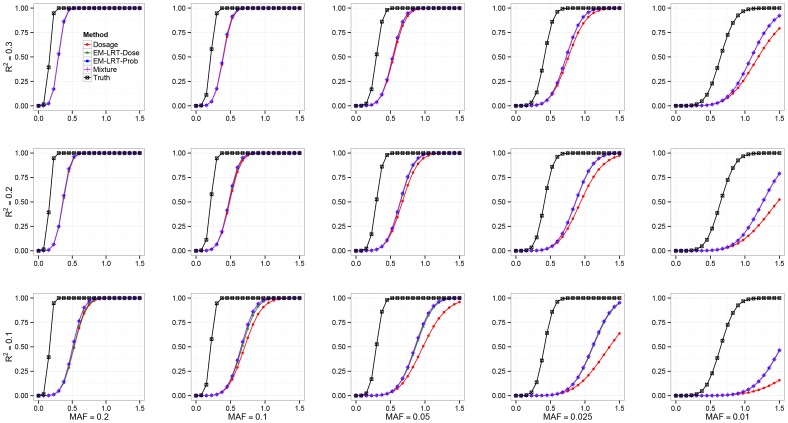
Power Comparison. The statistical power (Y-axis) of the different methods is shown across a spectrum of *R^2^* and MAF.

## Methods

We will first briefly review the Mixture method [13] given it is the state-of-the-art method that models the posterior probabilities. We will then proceed to introduce our methods by first introducing a hierarchical modeling framework to simulate genotype (including both the truth and imputed) and phenotype data of interest, explicitly taking imputation quality (as measured by *R^2^*, squared Pearson correlation between true and imputed genotypes) into account. This framework allows us to simulate data conditional on any desired *R^2^*, making it straightforward to evaluate performance at any desired level of imputation quality. We then describe our EM-LRT in two scenarios, namely when posterior probabilities are available (Scenario I) and when only imputed dosages are available (Scenario II).

### Brief Review of the Mixture Method

The following mixture of regression model is fit for trait *Y*_i_ and genotype probability vector 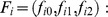




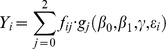
where 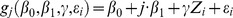, *Z*_i_ is the covariate vector, 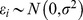, and *i* = 1, 2, …, *N* with *N* being the sample size. To estimate the parameters 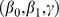, the log-likelihood function is maximized using the Nelder-Mead Simplex Method [25], implemented in R package *optim*.

### A Hierarchical Modeling Framework to Simulate Data

We adopt a hierarchical model that generates posterior probabilities, imputed dosages, and true genotypes using marker-specific information including minor allele frequency (MAF) and imputation quality measure (*R^2^*), as well as a quantitative trait with which we test for genetic association. The model has three stages: the first stage generates genotype probabilities based on marker-specific information (*genotype probability* stage); the second stage employs a multinomial distribution with probabilities from the first stage to generate allele counts (*allele count* stage); and the final stage fits a linear regression model to generate quantitative trait values (*trait* stage).

#### Genotype Probability Stage

For a specific marker with MAF *q* and imputation quality *R^2^*, the genotype probability vector 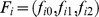 for the *i-*th sample is drawn from a Dirichlet distribution with parameters 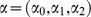, where 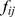 is the probability of having *j* copies of the minor allele for the *i-*th sample and 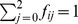. The parameters in the Dirichlet distribution are: 
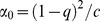
, 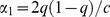, 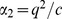 with 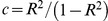. We provide details to justify this choice the parameters 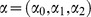 in Appendix S1. Here we give some brief explanations. First, this distribution gives reasonable expected values for 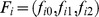 such that 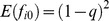, 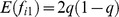, and 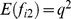, which are the expected probabilities of having 0, 1, or 2 copies of minor alleles assuming Hardy-Weinberg Equilibrium. Next, when *R^2^* approaches to 1, 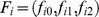 approaches to a distribution that takes three possible values, (1,0,0), (0,1,0), and (0,0,1) (i.e., the probability of having a particular genotype is either 0 or 1), which is the expected situation when there is no imputation ambiguity. Given the genotype probability vector, the imputed dosage is 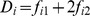.

#### Allele Count Stage

The allele count vector 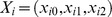 for the *i-*th sample is drawn from a multinomial distribution with genotype probabilities specified in the previous stage, where 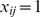 if the *i-*th sample has *j* copies of the minor allele; and 0 otherwise, with the constraint of 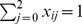. Additionally, the genotype *G_i_* for the *i-*th sample is generated using this allele count vector, specifically 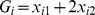. Our simulation framework, taking imputation quality *R^2^* into account using *c* above, renders 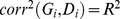 [proof in Appendix S1; Figure S2 and S3].

#### Trait Stage

In the final stage, a linear regression model is used to generate quantitative trait 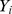 using genotype *G_i_* and covariates *Z_i._*

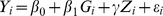
where 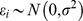 and *i* = 1, 2, …, *N*.

### Expectation-Maximization Likelihood-Ratio Tests

Our primary goal is to test for marker-trait association when marker genotypes *G* are not directly observed but rather imputed. We propose the following expectation-maximization likelihood-ratio tests (EM-LRT). We consider two common scenarios after genotype imputation: 1) when posterior probabilities of genotypes are available and 2) when only dosages are available.

#### Scenario I: When Posterior Probabilities Are Available [EM-LRT-Prob]

Under this scenario, the true genotype *G_i_* is missing but genotype probability vector 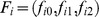 is estimated, *i* = 1, 2, …, *N* with *N* being the sample size. Given the observations 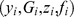 where 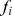 is the observed value for 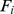, the complete data likelihood is.
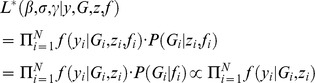
where the second equality holds because trait *y_i_* is independent of genotype probability vector *f_i_* conditional on true genotype *G_i_* and true genotype is independent of covariates *z_i_* conditional on genotype probability vector. Therefore, with Gaussian distribution, the corresponding complete-data log-likelihood is



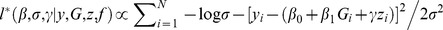



In this complete data log-likelihood, terms involving true genotype *G_i_*, namely *G_i_* and *G_i_^2^*, are not observed and will be replaced in the *E-step* by their conditional expectations given the observed data. Their conditional expectations are.
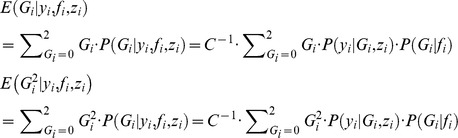
where 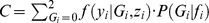, and 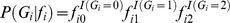.

In the *M-step*, the maximum likelihood (ML) estimates of the parameter 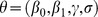 are obtained as follows:
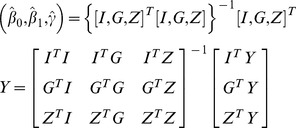





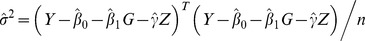
We repeat the *E-step* and *M-step* until convergence (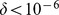).

To speed up the EM algorithm, we suggest using the naïve parameter estimates as starting values, that is, the parameter estimates derived by fitting a simple linear regression on trait *Y* using dosage *D* and covariates *Z* (a.k.a Dosage or standard method). Our EM-LRT-Prob approach shares some similarity with the seminar work by Lander and Botstein [26] for interval mapping, in which the authors also used mixture model framework, treating genotypes at quantitative trait loci as missing data.

#### Scenario II: When Only Dosages Are Available [EM-LRT-Dose]

We propose a framework that first uses the conditional (on dosages) distribution to sample genotype probabilities given the imputed dosages, and then apply the EM algorithm detailed above in Scenario I.

First, we derive the probability density function for 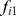, the probability of having one copy of the minor allele conditioning on imputed dosage.
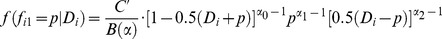
where *C′* is the normalizing constant, 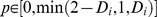, and *B(.)* is the beta function [Appendix S2]. Second, we select the envelope function 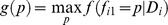 such that 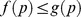 for all *p*. Third, we perform the following steps to sample 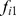: 1) generate 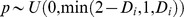; 2) generate 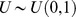; 3) accept *p* if 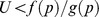. Finally, we calculate 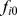 and 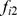 using the relationship 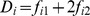 and 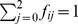.

The drawback of the above rejection sampling approach is that it can be computationally rather expensive especially when the envelope function is large. Fortunately, we can use an approximation approach when MAF is not high. For example, when MAF is low enough, the probability of having two copies of the minor allele is close to zero. In that case, we adopt an approximation approach (referred hereafter as dosage approximation approach) by setting the probability of having one copy of the minor allele to dosage when MAF is below certain threshold depending on the imputation quality [details are shown below in subsection **Numerical** **Simulation: MAF Threshold**].

### Hypothesis Testing

To assess whether a variant is associated with phenotypic trait of interest *Y*, we perform the following hypothesis testing 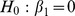 vs. 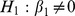. Note that the same 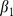 is assumed across all three possible genotypes. We propose to use the likelihood-ratio test for this purpose. Specifically, hypothesis testing is performed as follows: 1) use the EM algorithm described previously to find the ML estimates 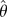 for 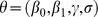, and then compute the log-likelihood 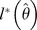; 2) find the ML estimates 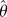 under *H_0_*; and 3) compute the likelihood-ratio statistics (*LRS*): 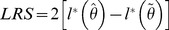. The LRT will reject the *H_0_* if 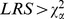, where 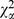 is the 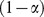
100^th^ percentile of the 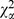-distribution with degree of freedom (d.f.) = 1.

### Numerical Simulation

#### MAF Threshold

To achieve optimal balance between performance and computational efficiency, we use extensive simulations to find the MAF threshold between the choice of rejection sampling and dosage approximation. Given *R^2^*, we calculate two sets of mean squared error (MSE) between sampled genotype probability 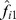 and truth 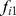 using rejection sampling and dosage approximation, respectively.

#### Type I Error Evaluation

We assess the validity of EM-LRT-Dose, Dosage, EM-LRT-Prob, Mixture and gold-standard (based on true genotypes) under various combinations of *R^2^* and MAF. Specifically, we simulate data sets each with 2,000 samples using pre-specified marker-specific information *R^2^* and MAF, which allows us to generate genotype probabilities, dosages, and true genotypes. Next, we simulate the trait values *Y_i_* according to the linear model for sample *i* with a set of pre-specified parameters, where *i* = 1, …, 2,000. For simplicity, we set 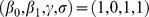.

We repeat the simulation ten million times. For each simulated data set, we perform association testing based on the true genotypes (truth), as well as based on imputed data using the standard Dosage method, Mixture method, and our proposed EM-LRT-Prob and EM-LRT-Dose methods. The empirical type I error of each method is calculated as the proportion of observed *p*-values that fall below the specified significance level. In addition, we calculate the Spearman correlation between the observed and gold-standard (true genotype based) *p*-values.

#### Statistical Power Assessment

To evaluate the statistical power of different methods, we again simulate data sets each with 2,000 samples using a combination of marker-specific information *R^2^* and MAF, and parameters 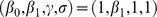 where 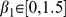. We again repeat the simulations one million times. Similarly, for each simulated data set, we performed the same set of tests. The power of each method is calculated as the proportion of observed *p*-values that fall below the significance threshold 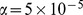.

## Results

### MAF Threshold

We used simulations to determine the MAF threshold specific to each *R^2^* such that the rejection sampling is advantageous (quantified by lower MSE in estimating 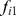, the probability of having one copy of the minor allele) over dosage approximation when exceeding the MAF threshold (Figure 1 and Table 1). We observed the two sampling methods have similar performance (measured by MSE) when MAF is not high (below 20%–30% depending on *R* ^2^). In such cases, we chose the simple dosage approximation method due to computational efficiency (Runtimes for rejection sampling and dosage approximation based on 2,000 samples are also shown). We also observed inferior performance (larger MSE) of both methods for low MAFs with intermediate *R* ^2^ values. Both can be explained by a combination of the imputation quality and the variation in 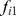 as a function of both imputation quality *R* ^2^ and MAF *q*. Specifically, we have Var(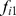) = *R* ^2^ × 2*q*(1– *q*) × (1–2*q*(1– *q*)), which increases with MAF *q* as well as with *R* ^2^. Low imputation quality *R* ^2^ coupled with low MAF leads to relatively little variation in 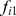, rendering both sampling methods capable of estimating it relatively accurately. On the other hand, high imputation quality implies dosages close to true genotype values 0, 1, and 2, as well as 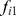 close to 0 or 1, thus allowing accurate inference of 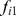 despite the larger variation in the values of 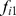 across individuals. In the intermediate *R* ^2^ range, variation in 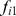 coupled with imputation uncertainty makes inference challenging for both approaches.

**Table 1:** Rejection Sampling vs. Dosage Approximation for 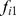 Estimation.

		Rejection Sampling		Dosage Approximation	
R^2^	MAF Cutoff	MSE	Runtime (Sec.)	_MSE_	Runtime (Sec.)
0.95	20%	1.01E-02	5.82	1.42E-02	7.60E-04
0.75	30%	4.49E-02	3.04	6.39E-02	6.80E-04
0.50	30%	5.98E-02	2.3	6.29E-02	7.14E-04
0.30	30%	5.71E-02	2.42	6.11E-02	8.10E-04
0.25	30%	5.32E-02	2.61	6.06E-02	8.36E-04
0.20	25%	3.82E-02	2.41	3.93E-02	7.56E-04
0.10	20%	1.69E-02	2.35	1.99E-02	7.04E-04

MAF: Minor allele frequency.

MSE: Mean square error.

### Empirical Type I Error Simulation Results

As shown in Table 2 (at significance level 5E-02) and Table 3 (at significance level 5E-05), all the methods have proper control of type I error across in the range of *R^2^* and MAF examined: 0.1≤ *R^2^*≤0.3 and 1% ≤ MAF ≤20%. Next, as shown in Figure 2, Spearman correlation with true *p*-values increases for every method when *R^2^* increases. The overall correlation is low in the range of MAF and *R* ^2^ examined. This low correlation is expected given the high level of imputation uncertainty and consistent with previous results [13], confirming that association inference is challenging with low frequency variants, or with variants imputed with a high level of uncertainty. Although the absolute performance of all methods is not particularly impressive, we observe that EM-LRT methods always show slightly higher Spearman correlation than Dosage method especially when MAF is low, suggesting that the EM-LRT *p*-values better approach gold-standard *p*-values. When *R^2^* and MAF are high, all methods perform similarly (results not shown), consistent with results shown in literature [11] [13].

**Table 2:** Type I Error at Significance Level = 5E-02.

R^2^	MAF	Dosage	EM-LRT-Dose	EM-LRT-Prob	Mixture	Truth
0.3	0.2	4.99E-02	5.01E-02	5.01E-02	5.01E-02	4.99E-02
	0.1	5.01E-02	5.02E-02	5.03E-02	5.03E-02	4.99E-02
	0.05	4.99E-02	5.01E-02	5.01E-02	5.01E-02	4.99E-02
	0.025	5.01E-02	5.03E-02	5.03E-02	5.03E-02	5.01E-02
	0.01	4.98E-02	4.96E-02	4.96E-02	4.96E-02	4.99E-02
0.2	0.2	5.00E-02	5.02E-02	5.03E-02	5.03E-02	5.00E-02
	0.1	4.99E-02	5.02E-02	5.03E-02	5.03E-02	5.00E-02
	0.05	5.00E-02	5.03E-02	5.03E-02	5.03E-02	5.01E-02
	0.025	4.99E-02	5.03E-02	5.03E-02	5.03E-02	5.01E-02
	0.01	5.00E-02	5.02E-02	5.01E-02	5.01E-02	4.99E-02
0.1	0.2	5.00E-02	5.08E-02	5.05E-02	5.05E-02	5.01E-02
	0.1	5.00E-02	5.06E-02	5.08E-02	5.08E-02	5.00E-02
	0.05	5.01E-02	5.11E-02	5.13E-02	5.13E-02	5.01E-02
	0.025	5.01E-02	5.15E-02	5.14E-02	5.14E-02	5.01E-02
	0.01	5.00E-02	5.09E-02	5.07E-02	5.07E-02	4.98E-02

**Table 3:** Type I Error at Significance Level = 5E-05.

R^2^	MAF	Dosage	EM-LRT-Dose	EM-LRT-Prob	Mixture	Truth
0.3	0.2	4.85E-05	5.00E-05	4.94E-05	4.94E-05	5.24E-05
	0.1	5.09E-05	4.98E-05	5.23E-05	5.23E-05	5.43E-05
	0.05	4.71E-05	5.03E-05	5.07E-05	5.07E-05	5.41E-05
	0.025	4.95E-05	4.92E-05	4.80E-05	4.80E-05	4.97E-05
	0.01	5.35E-05	4.79E-05	4.69E-05	4.69E-05	4.97E-05
0.2	0.2	4.58E-05	4.57E-05	4.58E-05	4.58E-05	5.00E-05
	0.1	4.67E-05	4.71E-05	4.84E-05	4.84E-05	5.30E-05
	0.05	5.08E-05	5.09E-05	5.05E-05	5.05E-05	5.14E-05
	0.025	5.23E-05	5.02E-05	5.09E-05	5.09E-05	5.06E-05
	0.01	4.78E-05	4.41E-05	4.26E-05	4.26E-05	4.92E-05
0.1	0.2	4.93E-05	5.53E-05	5.19E-05	5.19E-05	5.02E-05
	0.1	5.08E-05	5.27E-05	5.24E-05	5.24E-05	5.35E-05
	0.05	5.05E-05	5.09E-05	5.23E-05	5.22E-05	4.85E-05
	0.025	4.98E-05	5.15E-05	5.04E-05	5.04E-05	4.93E-05
	0.01	5.02E-05	5.05E-05	4.95E-05	4.95E-05	5.27E-05

### Empirical Power Simulations Results

When *R^2^* and MAF are high, all methods have similar performance. In this work, we focus on scenarios where 0.1≤ *R^2^*≤0.3 and 1% ≤ MAF ≤20%. As shown in Figure 3, EM-LRT-Prob and Mixture methods are consistently the most powerful methods among all methods evaluated. However, these methods are not applicable in scenario II when only imputed dosages are available. It is thus valuable to notice that EM-LRT-Dose method approaches the statistical efficiency of EM-LRT-Prob, outperformng the standard Dosage method especially when *R^2^* or MAF is low. For example, when *R* ^2^ = 0.1 and MAF = 0.05, the power for EM-LRT-Prob, Mixture, EM-LRT-Dose and Dosage are 84.5%, 84.5%, 82.1%, and 61.4% under *β*_1_ = 1.

### Application to CLHNS Data Set

We applied the proposed EM-LRT methods as well as other existing methods to the Cebu Longitudinal Health and Nutrition Survey (CLHNS) study of 1,800 unrelated Filipino women. We performed association analysis across chromosome 16, where we have previously identified the variants near *CDH13* gene associated with plasma adiponectin level [27].

We conducted association testing with standardized adiponectin level measured in 2005 on a log scale as the quantitative trait and adjusted for age and BMI also measured in 2005. Additionally, we excluded subjects from the analysis if they met one or more of the following criteria: 1) subjects with adiponectin level missing or outside of the range mean +/−4 standard deviations (n = 19); 2) subjects carrying the R221S variant (n = 53) [28]; and 3) subjects with missing age or BMI covariate information (n = 20). In total, 1,717 subjects were tested for association with adiponectin level.

These 1,717 subjects were genotyped on the Affymetrix Genomewide Human SNP Array 5.0 GWAS chip [29] and also on the Illumina HumanExome Beadchip. Specifically, we first established the truth by employing PLINK [30] to perform association on the true genotypes separately, finding 10 true positives (*p*-value <5×10^−6^) on Affymetrix 5.0 and 5 on exome chip (with 2 overlapping). Next, to mimic a setting of low imputation quality, we masked all neighboring GWAS SNPs within 2 kb of the 13 true positives before genotype imputation (22 SNPs were masked). Finally, we performed imputation using the MaCH imputation software [31] using the ASN panel from the Phase I 1000 Genomes Project (March 2012 release, version 3) as reference. To evaluate the performance of the proposed methods along with other alternatives, we used markers overlapping between the ASN reference panel and the exome chip, but not on the Affymetrix 5.0, at which we have both imputed genotypes and true genotypes (from exome chip genotyping). We then conducted association testing on the imputed genotypes (dosages or probabilities) using our proposed EM-LRT methods, Dosage, and Mixture method.

**Figure 4:**
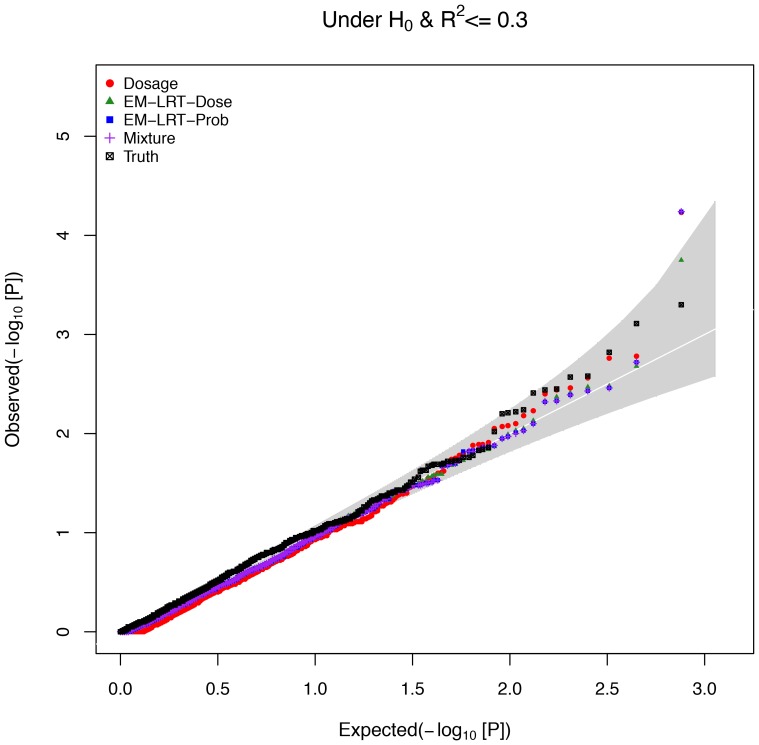
Q–Q Plot for Null Variants with Low Imputation Quality in the CLHNS Study. The observed (Y-axis) vs. expected (X-axis) –log_10_[*p*-values] are shown for 1,135 SNPs in the CLHNS data set. These SNPs are considered to be under the null hypothesis (true *p*-value >5×10^−6^), and all have low imputation quality (*R* ^2^<0.3).

Figure 4 shows the Q–Q plot for the 1,135 SNPs on chromosome 16 with *R^2^* ≤ 0.3 and true *p*-value >5×10^−6^. Q–Q plots are used to assess the number and magnitude of observed associations between tested SNPs and the trait under study, by comparing the observed –log_10_ *p*-values to what is expected under the null hypothesis of no association. Early departure from the identity line suggests either that there is uncontrolled confouding leading to false positives (for example, due to population stratification) or that a considerable proportion of SNPs are associated with the trait of interest (and thus not under the null distribution). Focusing on variants with *p*-values >5×10^−6^ based on experimental genotypes allowed us to examine the type I error empirically. Overall, this Q–Q plot suggests that all methods have proper control of type I error with all points falling within the 95% confidence bands with the exeption of one variant. The single potential false positive, rs8045889 with a true *p*-value = 0.0148; *R* ^2^ = 0.0736; and MAF = 0.4271, was identified by Dosage, EM-LRT-Prob, and Mixture (EM-LRT-Dose has a borderline *p*-value of 0.0002). In addition, we observe overall deflation in the test statistics (observed larger *p*-values) of all methods when compared with truth. The median (mean) *p*-values are 0.6407, 0.5614, 0.5568 and 0.5568 (0.6075, 0.5552, 0.5512, and 0.5543) for Dosage, EM-LRT-Dose, EM-LRT-Prob and Mixture respectively, compared with the true median (mean) of 0.5008 (0.5009). The tendency towards large *p*-values is expected and driven by the loss of information due to imputation uncertainty.

While establishing the validity is crucial, we are more interested in the power to identify genuine associations. Table 4 tabulates *p*-values from all four methods together with the truth for variants with *R* ^2^<0.3 and true *p*-value <5×10^−8^. Although all variants reach the genome-wide significance threshold regardless of the method, we observed that EM-LRT-Dose or EM-LRT-Prob generated more significant *p*-values (and better approached truth in all cases) than the alternatives for five out of the six variants interrogated, suggesting power enhancement using our methods.

**Table 4:** Associated Variants with *R^2^*≤0.3 in the CLHNS Study.

		*P*-values				
Coordinate*	*R* ^2^	Dosage	EM-LRT-Dose	EM-LRT-Prob	Mixture	Truth#
chr16∶82646152	0.251	**2.13E-11**	**1.45E-11** ^†^	4.68E-11	4.68E-11	6.77E-20
chr16∶82650717	0.282	**2.88E-11**	**1.83E-11** ^†^	5.87E-11	5.87E-11	1.35E-21
chr16∶82663288	0.268	**2.67E-10** ^†^	7.78E-10	**6.46E-10**	**6.46E-10**	2.16E-25
chr16∶82670249	0.270	2.04E-08	**1.45E-08** ^†^	**1.59E-08**	1.61E-08	1.72E-12
chr16∶82670539	0.249	9.26E-09	1.27E-08	**4.79E-09** ^†^	**4.83E-09**	1.25E-12
chr16∶82670636	0.230	1.22E-08	2.01E-08	**7.32E-09** ^†^	**7.33E-09**	1.78E-12

*: Coordinates are in genome build 37.

Bold with †: The most significant *p*-value among the four methods.

Bold without †: The second most significant *p*-values among the four methods.

# : Truth was established by regressing phenotype on true genotypes.

### Application to the Women’s Initiative Study of Blood Cell Traits

We have previously identified several variants associated with blood cell traits using whole exome sequencing in 761 African Americans coupled with imputation in >13,000 African Americans with GWAS data from genome-wide Affymetrix 6.0 genotyping [14]. The samples are drawn from several cohorts including WHI, ARIC, CARDIA and JHS. Association analyses were performed separately for WHI and CARe cohorts (ARIC, CARDIA and JHS) and subsequently meta-analyzed across the two. Due to the ascertainment of variants through whole exome sequencing, 56% of our analyzed variants had MAF <5%.

Here, we use meta-analysis results from our previous study as a gold standard to define true positives and investigate the *p*-values in the WHI cohort using our EM-LRT-Dose and standard Dosage method, which had been adopted by the original study. We did not keep a copy of the posterior probabilities because of the large number of samples imputed and because standard analyses do not involve the posterior probabilities. Therefore, this is a real data example of scenario II. Table 5 presents all variants with MAF <5% reported to reach genome-wide significant threshold in the original study, comparing *p*-values from our EM-LRT-Dose and the standard Dosage method. We notice that EM-LRT-Dose generated slightly more significant *p*-values at the associated variants in three out of the four tests performed. In one case (snp.177015 with white blood cell count [WBC]), the *p*-value from EM-LRT-Dose (*p*-value = 4.72×10^−8^) reached the conventionally employed genome-wide significance threshold of 5×10^−8^ while that from Dosage was marginally genome-wide insignificant (*p*-value = 6.11×10^−8^).

**Table 5:** Associated Variants with MAF <5% in the WHI Study.

			*P*-value	
SNP	Trait	Meta *p*-value	Dosage	EM-LRT-Dose
snp.684276	hematocrit	5.70E-11	5.85E-11*	8.94E-11
snp.177048	log(WBC)	3.00E-13	3.95E-08	2.73E-08*
snp.177015	log(WBC)	4.30E-13	6.11E-08	4.72E-08*
snp.41127	platelet	1.50E-11	2.52E-08	3.71E-09*

*: The most significant *p*-value among the two methods.

## Discussion

It is crucial to take imputation uncertainty into consideration when performing association testing. Existing methods have focused on common variants, which have been the focus of the past wave of GWAS using HapMap-based imputation. With the deluge of next generation sequencing data being generated, increasingly denser reference panels are allowing imputation of a much larger number of variants, including an increasing number of relatively rare or poorly imputed variants. It is thus highly warranted to re-visit potential strategies for post-imputation association analysis and to seek more powerful or efficient statistical methods.

In this work, we have proposed EM-LRT methods explicitly incorporating marker level imputation quality statistic into association tests. We considered two scenarios: when posterior probabilities of all potential genotypes are available and when only dosages are available. We evaluated the performance of the proposed methods along with existing alternatives using simulation studies and by application to real data sets.

**Figure 5:**
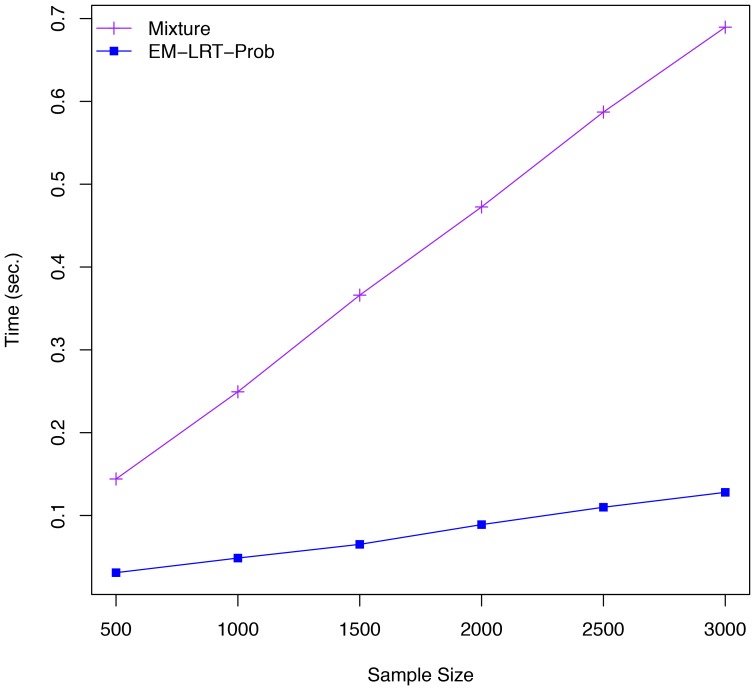
Computing Time: Mixture Method vs EM-LRT-Prob. The computing time of the Mixture method and our proposed EM-LRT-Prob method is displayed across a range of sample sizes. For each sample size, computing time is averaged across 2,000 simulated datasets.

In scenario I, our proposed EM-LRT-Prob demonstrated nearly identical performance as the Mixture model, which has been shown to be the best post-imputation association method particularly when imputation uncertainty is high [13]. While our EM-LRT-Prob effectively also fits a mixture model (therefore in terms of the underlying statistical model essentially the same as the Mixture method [13]), we have proposed and implemented a much more computationally efficient algorithm to fit the model. Mixture method used *R* function *optim*() to find ML estimates. Technically, the *optim*() function uses numerical differentiation to obtain ML estimates based on the score function and Hessian matrix, which is considerably slower than our proposed EM algorithm. To quantify the computational efficiency, we conducted association testing on a CLHNS data set of 1,717 subjects and 13,801 SNPs, using EM-LRT-Prob and Mixture methods with the same starting values (the Mixture method tends to run even slower without using the suggested starting values). We observed that the association tests required 279 seconds computing time and 0.91 GB RAM for EM-LRT-Prob and 1,505 seconds computing time and 1.23 GB RAM for the Mixture method on a 2.93 GHz Intel Xeon Processor X5670. Computing time scales linearly with sample size for both EM-LRT-Prob and the Mixture method (Figure 5).

In scenario II, the Dosage method has been shown analytically as the optimal one dimensional summary statistic for association testing in a typical linear model [11]. In this work, we extended the utility of this optimal one-dimensional measure by employing it together with the imputation quality measure *R^2^* first to sample posterior probabilities (in an attempt to rescue as much full information as possible) and then to conduct association testing on the sampled probabilities using our proposed EM-LRT method.

Our simulations suggest an advantage of the proposed methods over the standard Dosage method when imputation quality is relatively low, where imputation quality is measured by *R^2^*, the squared Pearson correlation between the imputed dosages and the unknown true genotypes. Since the calculation of *R^2^* requires true genotypes, it is not available in practice and imputation software provides an estimate based on the observed dispersion in imputed genotypes over its expected value. Such an estimate (Rsq in MaCH [31], MaCH-Admix [32], minimac [33]; R2 for BEAGLE [1] and INFO for IMPUTE/IMPUTE2 [34] [35]) has been widely used for the assessment of imputation quality and for post-imputation quality control. However, as shown in Figure S1 (based on the CLHNS data), MaCH Rsq is not a perfect measure of *R^2^*. For example, it has been reported earlier to have the tendency of underestimating true quality for common variants [36] [22]. We also observed the tendency of over-estimation towards the lower end of the MaCH Rsq. Therefore, we still recommend post-imputation quality filtering before application of our methods. We suggest application to variants with estimated *R* ^2^>0.1, which is less stringent than what is typically recommended [20] [22], but above which imputation quality is typically under- rather than over- estimated. To further examine the effectiveness of the filtering threshold, we quantified type I error via simulations for varying *R^2^* (four values examined: 0.05, 0.1, 0.3, 0.5) in combination with varying MAF (three values examined: 2.5%, 5% or 10%). Specifically, for each *R^2^* and MAF combination, we simulated A (A = 2500) (exchangeable) groups of data sets under the null hypothesis. For each group, we simulated B (B = 2000) data sets (again, under the null hypothesis) and calculated the *p*-values by applying all methods to each of the B = 2000 simulated datasets. We then calculated the group-specific type I error as the proportion of B = 2000 *p*-values (in that group) below the significance threshold of 0.05. We therefore obtained A = 2500 type I error estimates. Finally, we conducted the following one-sample t-test on these 2500 type I error estimates *H_0_*: type I error ≤0.05 vs. *H_1_*: type I error >0.05. Significant results from the t-test indicate inflated type I error. Results are shown in Table 6. As we can see the results suggest that the mixture model based methods (EM-LRT-Dose, EM-LRT-Prob, and Mixture) have inflated type I error when *R^2^*≤0.1, which is likely caused by the tendency of the mixture model over-fitting the data based on additional d.f. compared to the null model.

**Table 6:** One-sample T-test for Type I Error.

		*P*-values			
MAF	Method	*R^2^* = 0.05	*R^2^* = 0.1	*R^2^* = 0.3	*R^2^* = 0.5
0.025	Dosage	6.75E-01	2.72E-01	4.97E-01	7.33E-01
0.05		2.03E-01	6.38E-01	9.21E-01	1.88E-01
0.1		9.62E-01	8.75E-01	2.01E-01	6.78E-01
0.025	EM-LRT-Dose	8.96E-183*	3.00E-65*	2.00E-01	9.05E-01
0.05		6.15E-216*	5.11E-35*	3.89E-01	7.33E-03
0.1		1.40E-69*	9.73E-12*	4.73E-03	4.17E-02
0.025	EM-LRT-Prob	2.34E-111*	2.51E-55*	2.49E-01	8.69E-01
0.05		1.54E-174*	3.38E-40*	2.50E-01	4.81E-03
0.1		4.24E-134*	1.04E-24*	5.01E-04	2.91E-02
0.025	Mixture	5.45E-111*	3.72E-55*	2.52E-01	8.70E-01
0.05		2.94E-174*	4.79E-40*	2.55E-01	4.98E-03
0.1		7.60E-134*	1.22E-24*	5.30E-04	2.95E-02

*: *P*-value <5E-4.

In summary, we have proposed likelihood-ratio tests based on expectation maximization algorithms for post-imputation association testing. Simulation and real data analyses show our methods have protected type I error. In addition, simulation and real data results suggest slightly enhanced statistical power of our EM-LRT methods over a standard Dosage test, which has been shown to be the optimal one dimensional statistic for post-imputation association testing; and computationally more efficient (average more than fivefold reduction in computing time) than the Mixture method, which has been shown to be the most powerful at increased computational costs for variants imputed with high level of uncertainty. We anticipate our methods will replace the Mixture method for the analysis of low frequency variants or those imputed with high uncertainty. We envision our methods being applied on a larger scale for GWAS studies with imputation from sequencing based reference panels, including in the public domain, the 1000 Genomes Project [37] [18], the UK10K Project [38], and the reference haplotypes assembled by the International Haplotype Consortium [39], as well as study specific reference panels [22] [40] [14] [41] [20] [42] [43]. Our methods are implemented in software package EM-LRT, freely available at http://www.unc.edu/∼yunmli/emlrt.html.

## Supplementary Data

Supplemental Data, which include two appendixes and three figures, can be found with the article.

## Supporting Information

Figure S1 Estimated versus True Imputation Quality (Rsq vs. R^2^). The MaCH estimated imputation quality Rsq (Y-axis) is plotted against the true imputation quality *R^2^* (X-axis), which were calculated between genotype data from exome chip array and imputed genotype data (dosages). The red 45-degree line represents perfect estimation. A smooth density scatter plot is employed such that darker color corresponds to larger density and individual dots represent outliers.
(TIF)

Figure S2 Variance of Dosages vs. Variance of Genotypes. The variance of dosages (Y-axis) is plotted against the variance of genotypes (X-axis) computed using imputed dosages and genotype data from exome chip array in the CLHNS study. The red 45-degree line represents perfect correlation.
(TIF)

Figure S3 Boxplot of Observed *R^2^*. The observed *R^2^* (Y-axis) is shown across a spectrum of true *R^2^* (X-axis) and MAF.
(TIF)

Appendix S1 Simulation of data with desired imputation quality R^2^.
(PDF)

Appendix S2 Derivation of the probability density function for the probability of having one copy of the minor allele conditioning on dosage.
(PDF)
