# Supplementary figures and images for: Association Studies with Imputed Variants Using Expectation-Maximization Likelihood-Ratio Tests
Source: PLoS One. 2014 Nov 10;9(11):e110679. doi: 10.1371/journal.pone.0110679 (PMC4226494; doi:10.1371/journal.pone.0110679)

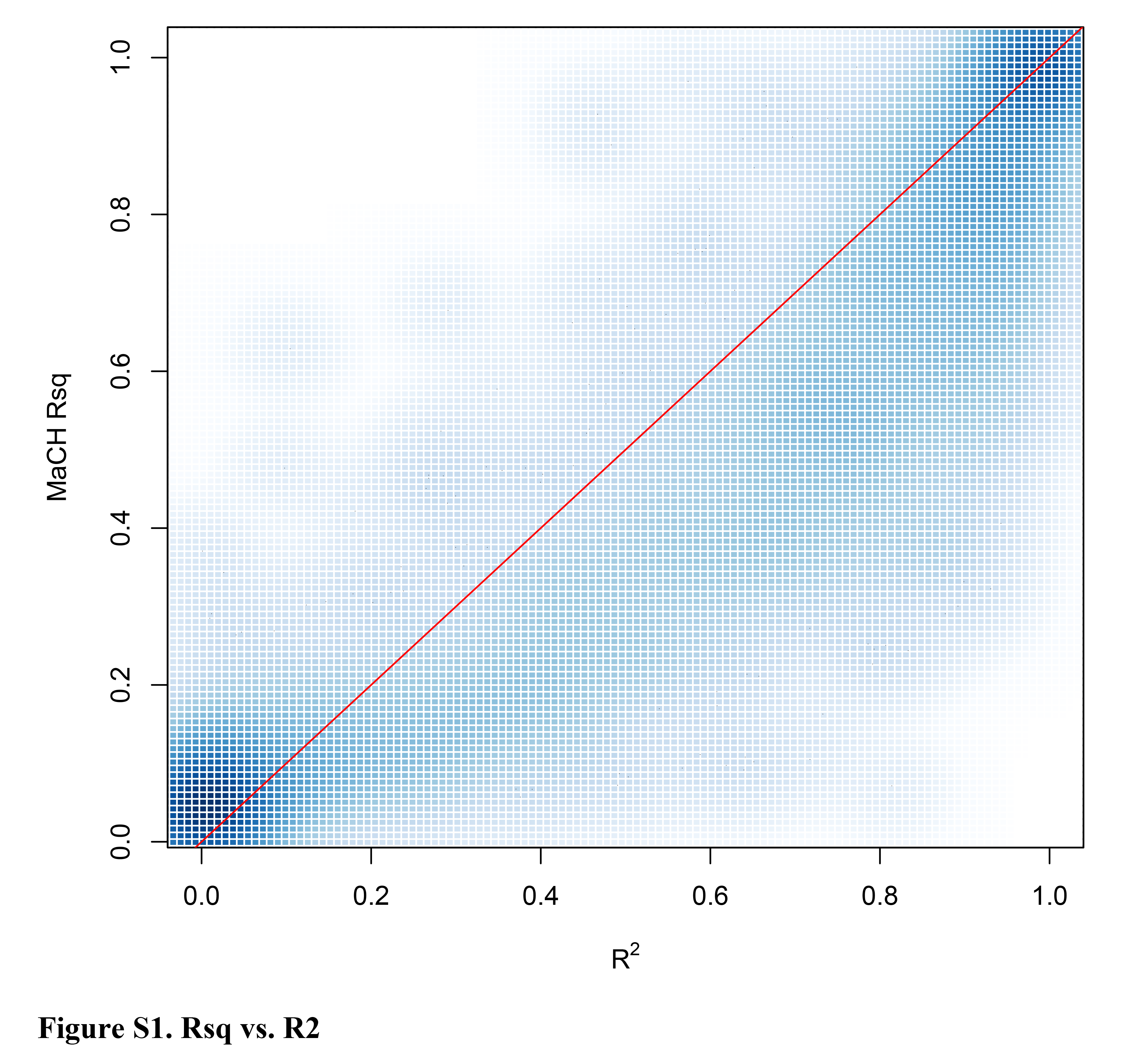

Supplement: Figure S1 — Estimated versus True Imputation Quality (Rsq vs. R2). The MaCH estimated imputation quality Rsq (Y-axis) is plotted against the true imputation quality R2 (X-axis), which were calculated between genotype data from exome chip array and imputed genotype data (dosages). The red 45-degree line represents perfect estimation. A smooth density scatter plot is employed such that darker color corresponds to larger density and individual dots represent outliers. (TIF) [file pone.0110679.s001.tif]

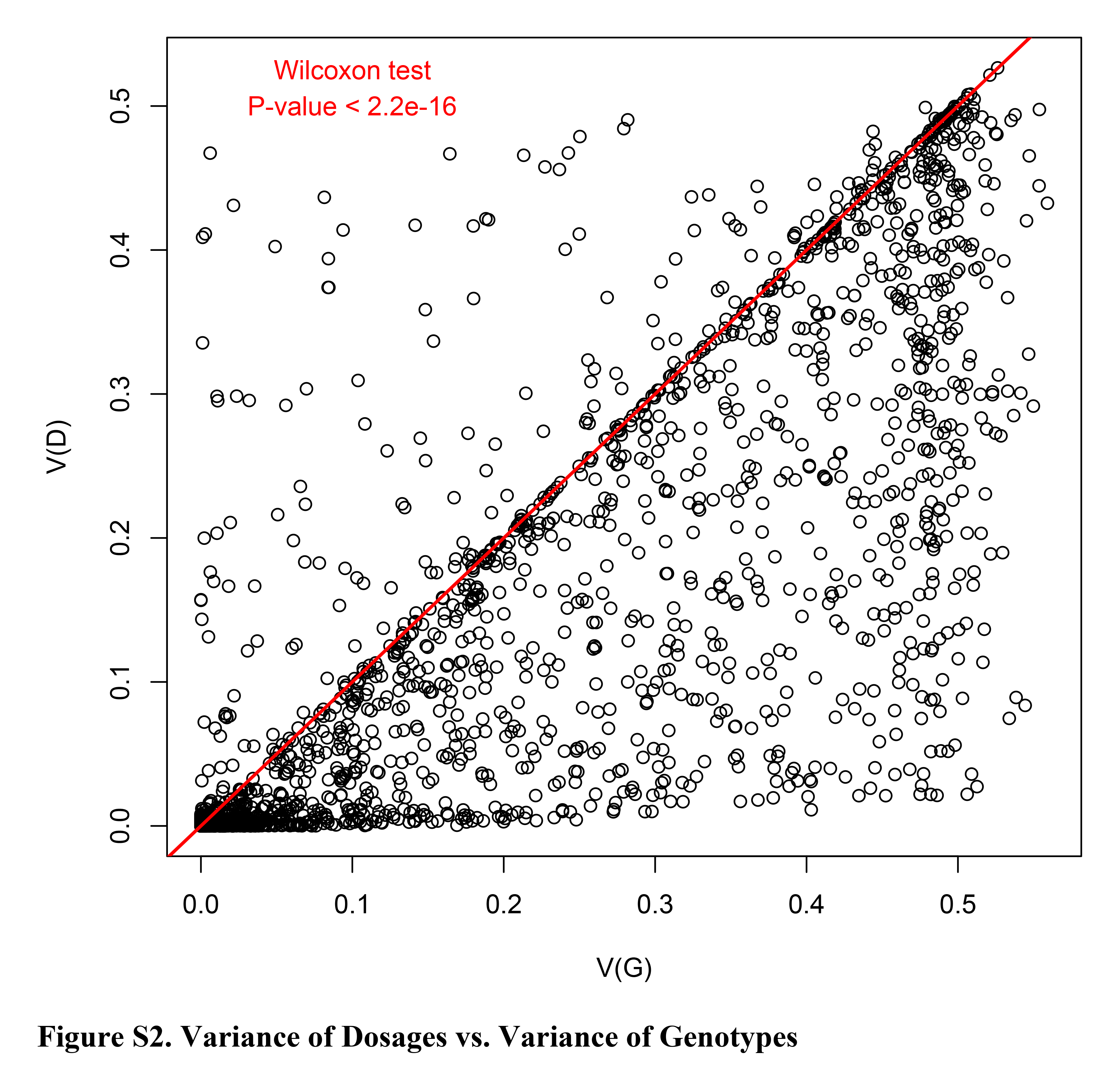

Supplement: Figure S2 — Variance of Dosages vs. Variance of Genotypes. The variance of dosages (Y-axis) is plotted against the variance of genotypes (X-axis) computed using imputed dosages and genotype data from exome chip array in the CLHNS study. The red 45-degree line represents perfect correlation. (TIF) [file pone.0110679.s002.tif]

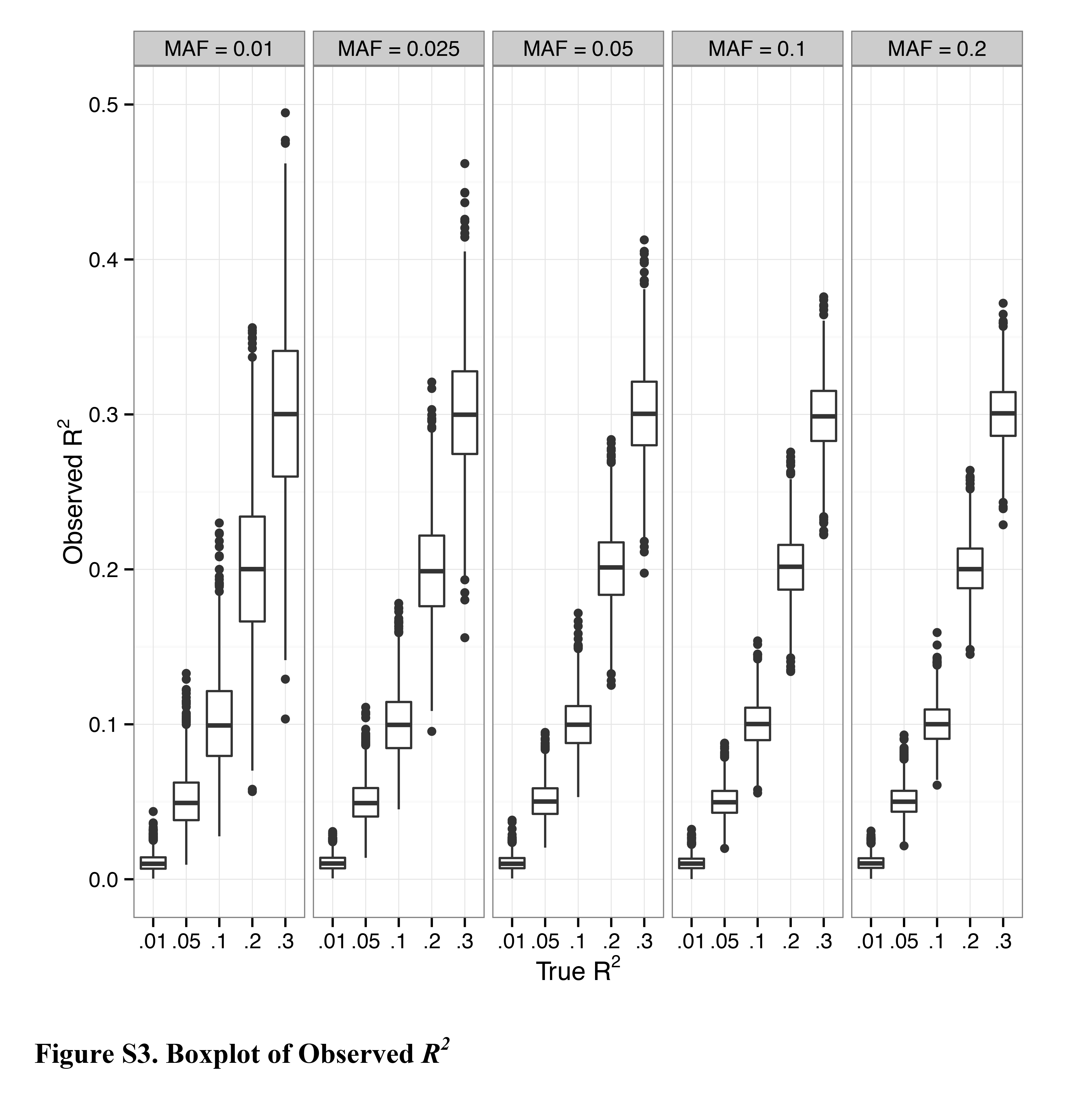

Supplement: Figure S3 — Boxplot of Observed R2. The observed R2 (Y-axis) is shown across a spectrum of true R2 (X-axis) and MAF. (TIF) [file pone.0110679.s003.tif]
