# Supplementary material for: Association Studies with Imputed Variants Using Expectation-Maximization Likelihood-Ratio Tests
Source: PLoS One. 2014 Nov 10;9(11):e110679. doi: 10.1371/journal.pone.0110679 (PMC4226494; doi:10.1371/journal.pone.0110679)
Supplement: Appendix S1 — Simulation of data with desired imputation quality R2. (PDF) [file pone.0110679.s004.pdf]

Appendix S1: Simulation of data with desired imputation quality R^2

Given the genotype probability vector $F_i = (f_{i0}, f_{i1}, f_{i2})$ and allele count vector X_i $= (x_{i0}, x_{i1}, x_{i2})$, the true genotype and dosage are then defined as $G_i = x_{i1} + 2x_{i2}$ and $D_i = f_{i1} + 2f_{i2}$. We assume, for the *i-th* subject, the genotype probability vector follows a Dirichlet distribution, $F_i \sim Dirichlet(\alpha)$, with parameter $\alpha = \left((1-q)^2/c, 2q(1-q)/c, q^2/c\right)$ where $c = R^2/(1-R^2)$. Given F_i, the allele count vector follows Multinomial distribution, $X_i \mid F_i \sim Multinomial(1, F_i)$. The above distributions for F_i and $X_i \mid F_i$ are sufficient for our following derivations. To elucidate the relation between X_i and F_i, we further derive the distribution of X_i and $F_i \mid X_i$. The marginal distribution of X_i ($\int$ P($F_i \mid X_i$) P(F_i) dF_i) is a Dirichlet-multinomial distribution. In this case where $x_{i0} + x_{i1} + x_{i2}$=1, it reduced to a multinomial distribution: $X_i \sim Multinomial((1\text{-q})^2, 2\text{q}(1\text{-q}), \text{q}^2)$. Then we can derive the conditional distribution of $F_i \mid X_i$ by the Bayes rule, which turns out to be *Dirichlet*(α'), where $\alpha' = (\text{x}_{i0} + (1\text{-q})^2/\text{c}, \text{x}_{i1} + 2\text{q}(1\text{-q})/\text{c}, \text{x}_{i2} + \text{q}^2/\text{c})$.

Now, since dosages range continuously from 0 to 2, the variability of dosages tends to be less than that of true genotypes. For example, in our Cebu Longitudinal Health and Nutrition Survey (CLHNS) study, we first calculated, for each marker, the variance of genotypes from exome chip genotyping and the variance of imputed dosages (dataset and imputation detailed in under Results subsection Application to CLHNS Dataset). Next, we conducted a Wilcoxon signed-ranked test with the hypothesis $H_0: V(G) = V(D)$ vs. $H_1: V(G) > V(D)$. The resulting p-value < 2.2e-16 leads us to reject the null hypothesis and conclude that the variability of dosages is less than that of

true genotypes (Figure S2). In addition, dosage is an expected allele count. It is thus reasonable to assume an unbiased Berkson error model: $G_i = D_i + \varepsilon_i$, where ε_i has mean zero and independent from D_i.

Next, we derive the variance of true genotype and dosage separately and then show its connection with imputation quality. By law of total variance, $V(G_i) = V\left[E(G_i|F_i)\right] + E\left[V(G_i|F_i)\right] = V(D_i) + E\left[V(G_i|F_i)\right]$, which gives an analytical underpinning behind the observation that genotype variability is no smaller than dosage variability given the genotype probabilities because $E\left[V(G_i|F_i)\right] \geq 0$. Further, $V(D_i) = V(f_{i1} + 2f_{i2}) = V(f_{i1}) + 4V(f_{i2}) + 4Cov(f_{i1}, f_{i2}) = \left[c/(1+c)\right]2q(1-q)$ and $E\left[V(G_i|F_i)\right] = E\left[V(x_{i1} + 2x_{i2}|F_i)\right] = E\left[V(x_{i1}|F_i) + 4V(x_{i2}|F_i) + 4Cov(x_{i1}, x_{i2}|F_i)\right] = \left[1/(1+c)\right]2q(1-q)$. Taken together, we have $V(G_i) = 2q(1-q)$. Last, we show that imputation quality can be controlled by *c* because

$$R^2 = \frac{Cov(G_i, D_i)^2}{V(G_i) \cdot V(D_i)} = \frac{V(D_i)}{V(G_i)} = \frac{c}{1+c}$$

where the second equality holds because $Cov(G_i, D_i) = Cov(D_i + \varepsilon_i, D_i) = V(D_i)$.

To confirm the validity of our model, we simulated the dataset with 2,000 samples across the spectrum of R^2 and MAF. We ran the simulation 2,000 times, and for each simulated dataset, we recorded the observed correlation between true genotypes and dosages. Figure S3 shows the boxplot of observed R^2 compared to true R^2 and MAF. From the figure, we notice that the observed R^2 has a median (as well as a mean) that is very close to the true R^2, illustrating the utility of our simulation framework to generate data given marker MAF and imputability.
