# Supplementary material for: Association Studies with Imputed Variants Using Expectation-Maximization Likelihood-Ratio Tests
Source: PLoS One. 2014 Nov 10;9(11):e110679. doi: 10.1371/journal.pone.0110679 (PMC4226494; doi:10.1371/journal.pone.0110679)
Supplement: Appendix S2 — Derivation of the probability density function for the probability of having one copy of the minor allele conditioning on dosage. (PDF) [file pone.0110679.s005.pdf]

Appendix S2: Derivation of the probability density function for the probability of having one copy of the minor allele conditioning on dosage

We assume, for the *i-th* subject, genotype probability vector follows Dirichlet distribution, $F_i = (f_{i0}, f_{i1}, f_{i2}) \sim Dirichlet(\alpha)$, with parameter $\alpha = (\alpha_0, \alpha_1, \alpha_2)$. The dosage is then defined as $D_i = f_{i1} + 2f_{i2}$ which yields $f_{i2} = 0.5(D_i - f_{i1})$ and $f_{i0} = 1 - f_{i1} - f_{i2} = 1 - 0.5(D_i + f_{i1})$. Therefore, after replacing f_{i0} and f_{i2} in the $f(f_{i0}, f_{i1}, f_{i2}; \alpha_0, \alpha_1, \alpha_2)$, we rewrite the function as

$$f(f_{i1}, D_i; \alpha_0, \alpha_1, \alpha_2) = \frac{1}{B(\alpha)} \left[1 - 0.5(D_i + f_{i1})\right]^{\alpha_0 - 1} f_{i1}^{\alpha_1 - 1} \left[0.5(D_i - f_{i1})\right]^{\alpha_2 - 1}$$

where *B(.)* is the beta function; $1 - 0.5(D_i + f_{i1})$, f_{i1}, and $0.5(D_i - f_{i1}) \in [0,1]$, which can be further simplified to $f_{i1} \in [0, \min(2 - D_i, 1, D_i)]$. Next, the conditional probability of f_{i1} and D_i can be derived as follows

$$f(f_{i1} | D_i) = \frac{f(f_{i1}, D_i)}{f(D_i)} = \frac{C'}{B(\alpha)} \left[1 - 0.5(D_i + f_{i1})\right]^{\alpha_0 - 1} f_{i1}^{\alpha_1 - 1} \left[0.5(D_i - f_{i1})\right]^{\alpha_2 - 1}$$

where $C' = f(D_i)^{-1} = \left[\int f(f_{i1}, D_i) df_{i1}\right]^{-1}$ is the normalizing constant.
